# Identification of Prognostic Biomarkers in Gene Expression Profile of Neuroblastoma Via Machine Learning

**DOI:** 10.1002/pdi3.70009

**Published:** 2025-05-27

**Authors:** Shuxin Tang, Jinhua Fan, Yupeng Cun

**Affiliations:** ^1^ Chongqing Key Laboratory of Child Neurodevelopment and Cognitive Disorders Ministry of Education Key Laboratory of Child Development and Disorders Pediatric Research Institute National Clinical Research Center for Child Health and Disorders Children's Hospital of Chongqing Medical University Chongqing China

**Keywords:** gene expression profile, machine learning, molecular network, neuroblastoma, prognostic biomarkers

## Abstract

Neuroblastoma (NB) is a common pediatric solid malignancy characterized by heterogeneous clinical outcomes. The identification of predictive and interpretable prognostic biomarkers is critical for advancing precision medicine in NB. We proposed an integrative network‐based machine learning method for biomarker discovery, which employed a network smoothed *t*‐statistic support vector machine to select prognostic related biomarkers, and then we performed network analysis on these biomarkers to find hub genes. Later, we conducted a comprehensive analysis to integrate bulk and single‐cell RNA sequencing data to character the tumor microenvironment of prognostic state and correlated them to the discovered hub genes. This analysis identified 528 prognostic biomarkers associated with NB. Network‐based analysis further refined this set to 11 hub prognostic biomarkers for NB: *AURKA*, *BLM*, *BRCA1*, *BRCA2*, *CCNA2*, *CHEK1*, *E2F1*, *MAD2L1*, *PLK1*, *RAD51*, and *RFC3*. Among these genes, high *RFC3* expression was significantly associated with poor prognosis, highlighting its potential as a novel prognostic biomarker in NB. Additionally, our findings revealed that these biomarkers are correlated to chemotherapy drugs, such as vincristine and cyclophosphamide. Furthermore, drug sensitivity analyses identified several candidate drugs, such as dactinomycin, bortezomib, docetaxel, and sepantronium bromide, that may hold therapeutic potential for NB treatment. This study offers novel insights to underlying NB prognosis and therapeutic targets and provides a foundation for developing personalized treatment strategies to improve clinical outcomes.

## Graphical abstract

This study identified 11 core prognostic biomarkers for neuroblastoma through optimized PPI networks and machine learning, revealing their correlations with chemotherapy and the tumor microenvironment, providing new insights for precision treatment and target development.

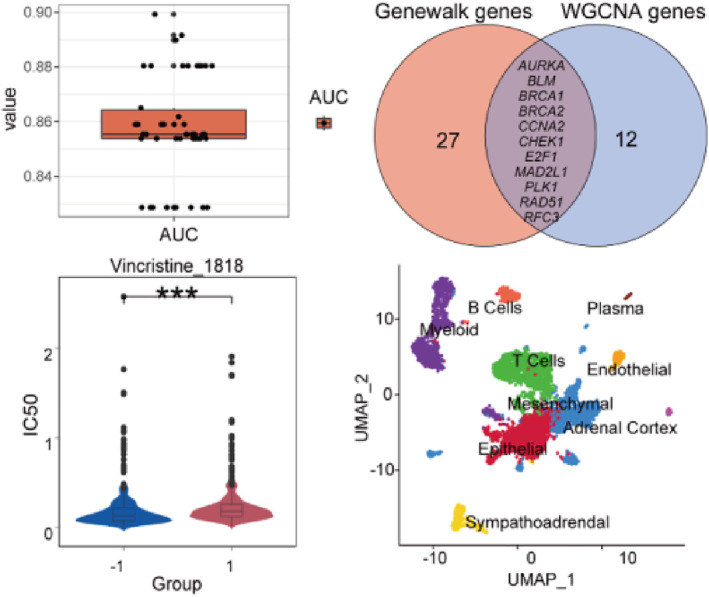

## 1 Introduction

Neuroblastoma (NB), a pediatric neuroendocrine tumor, is the most common solid malignant tumor diagnosed in early childhood [1]. Accounting for 8%–10% of all pediatric cancers, NB is one of the most aggressive and lethal pediatric malignancies tumor, responsible for 15% of all childhood cancer‐related deaths [2]. The median age of diagnosis is 19 months, underscoring the early onset of the disease [3]. Despite advancements in multimodal therapies, including chemotherapy, radiation, stem cell transplantation, and immunotherapy, the overall 5‐year survival rate remains below 60% [4].

Biomarkers are critical in early cancer diagnosis, prognosis evaluation, and the development of targeted therapies [5]. High‐throughput omics technologies and machine learning have revolutionized biomarker discovery across various diseases [5, 6]. In NB, biomarkers are essential for accurate diagnosis, prognostic evaluation, and the design of personalized therapeutic strategies. Among these, *MYCN* amplification is the most well‐established biomarker, strongly associated with poor prognosis, particularly in high‐risk patients [7]. Other biomarkers, such as *ALK* and *PHOX2B* mutations and *TERT* activation, also contribute to prognostic evaluations [8, 9, 10]. However, the predictive power of these known biomarkers is limited. For instance, *MYCN* amplification is present in only 25% of patients [7], *ALK* mutations are detected in only 7%–10% of cases [9], and *TERT* activation often necessitates complex gene rearrangement analyses [10]. Similarly, *PHOX2B* mutations, although relevant in familial NB, are rarely observed in sporadic cases [8]. Given these limitations, there is an urgent need to explore new prognostic biomarkers to enhance diagnostic accuracy, refine risk stratification, and improve treatment outcomes.

**FIGURE 1:**
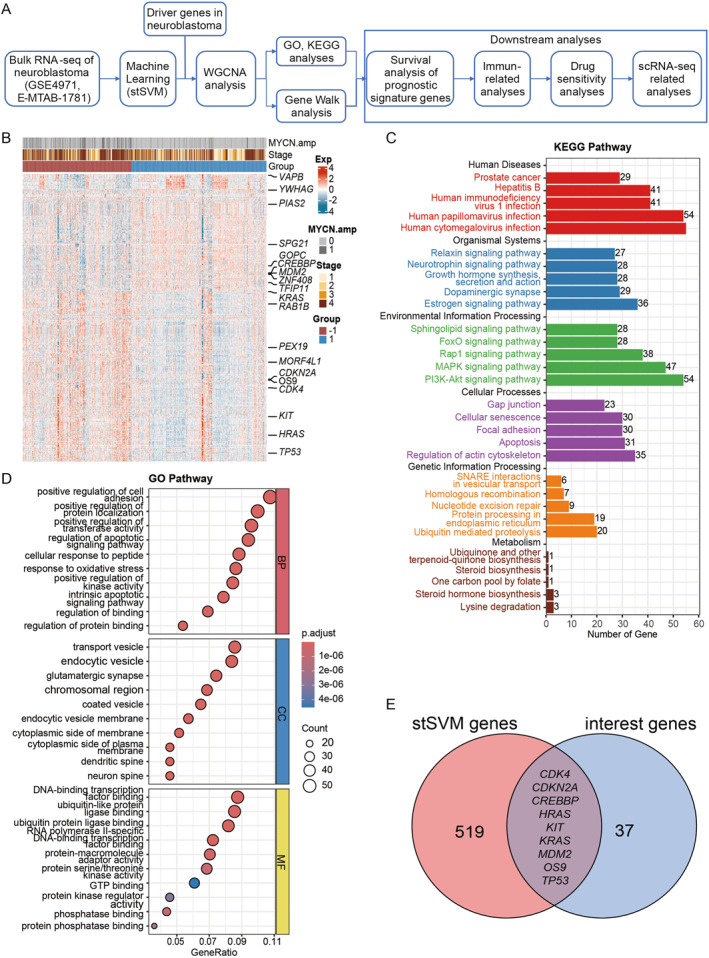
Prognostic biomarkers discovery pipeline via machine learning. (A) Workflow of NB prognostic biomarker discovery and related downstream analyses. (B) Heatmap showing gene expression patterns of prognostic biomarkers derived through machine learning. (C) KEGG pathway enrichment analysis of biomarkers. (D) GO pathway enrichment analysis of biomarkers. (E) Venn diagram showing overlap between machine learning‐derived prognostic biomarkers and driver genes identified in the literature. GO, gene ontology; KEGG, Kyoto Encyclopedia of Gene Genomes; NB, neuroblastoma; scRNA‐seq, single‐cell RNA sequencing; WGCNA, weighted gene co‐expression network analysis.

In this study, we performed a comprehensive analysis of bulk RNA sequencing (RNA‐seq) data from 1207 patients with NB across two datasets and single‐cell RNA sequencing (scRNA‐seq) data from 10 patients with peripheral neuroblastic tumor (PNT) (Figure 1A) [11, 12, 13]. We presented an enhanced version of our previously developed feature selection method, network smoothed *t*‐statistic support vector machine (stSVM) [14, 15], to identify prognostic biomarkers associated with NB. Subsequent network analysis refined this list, revealing 11 hub prognostic biomarkers: *AURKA*, *BLM*, *BRCA1*, *BRCA2*, *CCNA2, CHEK1*, *E2F1*, *MAD2L1*, *PLK1*, *RAD51*, and *RFC3*. Among these, *RFC3* was identified as a novel prognostic biomarker. To address the limitations of previously established biomarkers, we employed network biology approaches to identify candidates with enhanced prognostic and therapeutic relevance. This network‐based integration pipeline provides an efficient way to uncover biomarkers for prognosis prediction and inform therapeutic strategies for NB.

## 2 Materials and Methods

### 2.1 Sample Collection

Gene expression data from 1207 patients with NB, including overall survival time and status, were obtained from two public datasets (GSE49710 and E‐MTAB‐1781) [11, 12]. Batch effects were removed from the datasets using the *removeBatchEffect* function in limma (v3.58.1) [16]. The 5‐year survival rate was used as a critical indicator for the prognosis assessment. The combined gene expression data were categorized into two groups: patients with an overall survival time of less than 5 years and those with an overall survival time of 5 years or more. Additionally, scRNA‐seq data (GSE192906) [13] was employed for comprising survival time and status. A summary of these datasets was presented in Table 1.

**TABLE 1:** Overview of three NB datasets.

ID/source	Data type	Patient number	Cancer type	Classification	Positive class
GSE49710	Bulk	498	NB	≥ 5 years	269
E‐MTAB‐1781	Bulk	709	NB	≥ 5 years	398
GSE192906	Single cell	10	PNT	> 3 years	2

Abbreviations: NB, nueroblastoma; PNT, peripheral neuroblastic tumors.

### 2.2 Improved Network stSVM

StSVM algorithm, with *cv. stsvm* function in net Class (v1.2.1) [14, 15], was enhanced by added *logFoldChange* function of limma (v3.58.1) [16] to increase the rigor of gene selection. The predictive performance of the model was assessed using 10 times repetitions with 5‐fold cross‐validation. Prior to machine learning training, gene expression data were standardized and the protein–protein interaction (PPI) network was preprocessed in accordance with previous research [14]. Complex heatmaps of feature genes were generated using ComplexHeatmap (v2.18.0) [17].

### 2.3 Co‐Expression Network Analysis

Co‐expression network analysis was constructed via weighted gene co‐expression network analysis (WGCNA, v1.72.5) [18] to identify prognosis related modules. Hierarchical clustering of gene expression data were performed using *hclust* function. Optimal soft threshold value for network construction, based on approximate scale‐free topology, was determined using the *pickSoftThreshold* function. Gene co‐expression modules were constructed using the *blockwiseModules* function, and clustering analysis grouped genes into distinct modules, which were color‐coded for visualization using the *plotDendroAndColors* function. Module eigengenes (MEs) were calculated to assess correlations with phenotypes, and heatmaps of ME‐phenotype correlations were visualized using the R function *labeledHeatmap*. Hub genes within the network were identified through minimum spanning tree‐based approaches, as outlined in our previous studies [19, 20], and visualized via Cytoscape (v3.10.2) [21].

### 2.4 Enrichment Analysis

Gene ontology (GO) and Kyoto Encyclopedia of Gene Genomes (KEGG) analyses were performed on feature genes obtained from improved stSVM and the WGCNA modules using clusterProfiler (v4.10.1) [22]. Gene IDs were processed and converted using the bitr function in stringr package (v1.5.1) [23]. GO enrichment analysis focused on biological processes (BPs), cellular components (CCs), and molecular functions (MFs), using the enrichGO function. Top five KEGG pathways with the lowest *p*‐values for the stSVM feature genes were extracted for each category and grouped accordingly. Bar plots displaying gene counts, pathway descriptions, and categories were generated using ggplot2 (v3.5.0) [24]. WGCNA module genes were mapped to cancer‐related KEGG pathways, and a chord diagram illustrating gene‐pathway interactions was generated with the *GOChord* function in GOplot package (v1.0.2) [25]. GO analysis results were visualized using the *dotplot* function from enrichplot (v1.22.0) [26].

An in‐depth analysis of module genes associated with survival time from the WGCNA was conducted using GeneWalk (v1.6.2) [27]. The “id_type” parameter was set to “hgnc_symbol” to ensure the use of HGNC‐standard gene symbols. GO annotations were integrated to identify key regulatory genes with high connectivity and relevance to other input genes. Final set of hub prognostic biomarkers was derived from the intersection of hub genes identified using GeneWalk and the WGCNA survival‐associated modules.

### 2.5 Survival Analysis

Survival differences between high‐ and low‐expression groups, stratified by median gene expression, were analyzed using survival (v3.5.8) [28]. Survival curves for each group were fitted with the *survfit* function, and the Wilcoxon test was applied to evaluate survival differences, with corresponding *p*‐values calculated to assess significance. Kaplan–Meier survival curves were generated using the *ggsurvplot* function, with customized graphic parameters.

### 2.6 Immune‐Related Analysis

Relative proportions of different cell types were inferred from bulk RNA‐seq data using the xCellAnalysis function in xCell (v1.1.0) [29]. Boxplots comparing tumor microenvironment, immune, and stromal scores between the two groups with different survival times were generated using ggplot2 (v3.5.0) and ggpubr (v0.6.0) [30]. The composition of immune cell types was visualized using multibox plots. Estimation of Stromal and Immune Cells in Malignant Tumor Tissues Using Expression Data (ESTIMATE) [31] and Microenvironment Cell Populations‐counter (MCPcounter) [32] algorithms were employed to quantify immune cell subsets.

### 2.7 Drug Sensitivity Analysis

Drug response predictions were performed using oncoPredict (v1.2) [33], evaluating the sensitivity of NB samples to specific drugs and identifying correlations with biomarkers. GDSC2 dataset from the Genomics of Drug Sensitivity in Cancer (GDSC) database served as the training set. Gene expression data were input into the model, and drug responses were predicted for each sample using the *calcPhenotype* function. Statistical differences in predicted drug responses were assessed using the Wilcoxon rank‐sum test. Violin plots of key drugs were generated using ggplot2 (v3.5.0) and ggpubr (v0.6.0), highlighting statistical significance. Pearson correlations between gene expression and drug sensitivity were calculated using the *cor* function, revealing potential relationships between key genes and specific drugs.

### 2.8 ScRNA‐Seq Analysis

ScRNA‐seq data were processed using Seurat (v5.0.3) [34]. Raw unique molecular identifier (UMI) count data were converted to Seurat objects using the *CreateSeuratObject* function. Sample integration was achieved using *FindIntegrationAnchors* and *IntegrateData* functions. Data were filtered to retain cells expressing more than 200 genes, while excluding cells associated with noncoding genes. Doublets were removed using DoubletFinder (v2.0.4) [35], resulting in a final dataset of 5358 cells for further analysis.

In total, 3000 highly variable genes were selected using the *FindVariableFeatures* function, followed using normalization of the gene expression data. Principal component analysis (PCA) was conducted, with Uniform Manifold Approximation and Projection (UMAP) used for further visualization. Cell clustering was performed using *FindNeighbors* and FindClusters functions. Cells were annotated based on marker genes and the original articles of the referenced single‐cell data [13]. UMAP visualizations were customized with the *DimPlot* function. Proportions of different cell types were plotted using bar charts, and average expression values for prognostic markers were calculated using the *AverageExpression* function. Heatmaps of gene expression across samples were generated using the *pheatmap* function, and UMAP plots were created for selected subsets.

## 3 Results

### 3.1 Identification of NB Prognostic Gene Signatures Using Machine Learning

Gene expression data from 1207 patients with NB were retrieved from the GSE49710 and E‐MTAB‐1781 public databases [11, 12], with batch effects removed using the *removeBatchEffect* function in the limma. Patients were categorized into a poor prognosis group (overall survival < 5 years, marked as −1) and a favorable prognosis group (overall survival ≥ 5 years, marked as 1) (Supporting Information S1: Figure S1A). To identify prognostic biomarkers associated with NB, an improved stSVM algorithm was applied, which integrated biological network data and significantly refined feature selection precision within the machine learning framework [15, 16]. By incorporating PPI network into the gene expression matrix during feature training, 528 prognostic biomarkers were identified, with a median area under receiver operating characteristic curve (AUC) value of 0.855 (Figure 1B, Supporting Information S1: Figure S1B). Key biomarkers identified through machine learning training, such as *HRAS* and *TP53*, are known driver genes in NB [36], highlighting their potential contributions to disease progression.

To explore the biological functions and roles of the identified prognostic biomarkers, KEGG and GO pathway enrichment analysis were performed. Notably, theses prognostic‐related genes were significantly enriched in PI3K‐Akt and MAPK signaling pathways and related to cell proliferation, differentiation, and signal transduction (Figure 1C) [37, 38]. GO analysis further revealed significant enrichment in cell adhesion, vesicle transport, and protein binding/regulation pathways (Figure 1D). These findings suggest that the identified prognostic biomarkers were involved in key biological processes underlying the NB progression.

### 3.2 NB Prognostic Related Network Module During the Co‐Expression Network Analysis

To explore the relationship between machine learning‐identified prognostic biomarkers and known NB driver genes, 46 NB driver genes were extracted from the literature and integrated with prognostic feature genes for further analysis [36, 39] (Supporting Information S1: Table S1). Nine of the selected prognostic biomarkers overlapped with the NB driver genes (Figure 1E), which include well‐known NB driver genes, such as *TP53* and *KRAS*, and highly expressed in the poor prognosis group. These findings suggest that these genes may play crucial roles in NB prognosis [40, 41].

**FIGURE 2:**
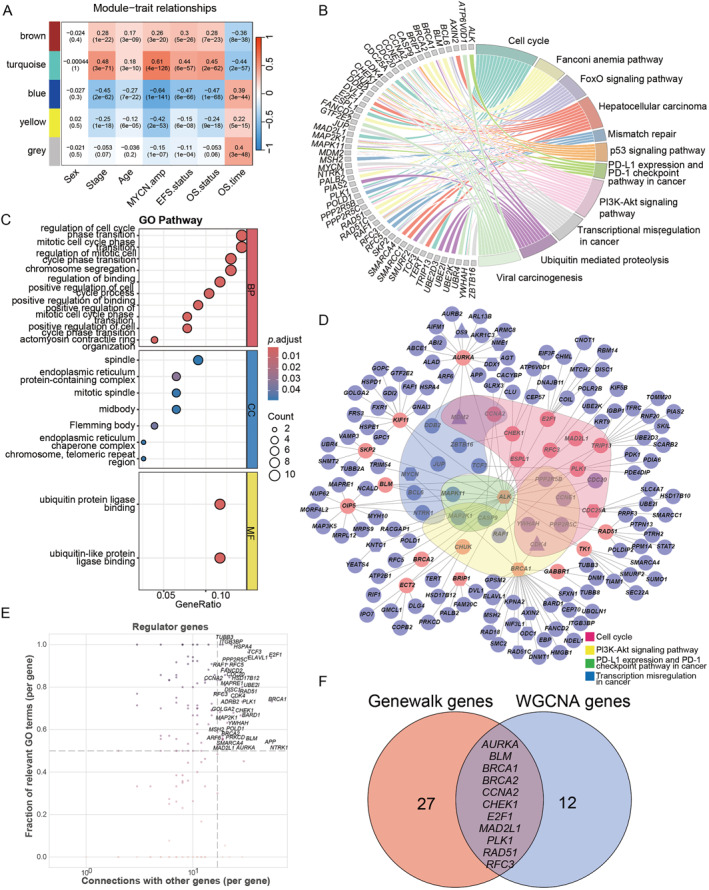
Identification of key prognostic biomarkers. (A) Relationships between gene modules and key traits by WGCNA. *MYCN*. amp: *MYCN* amplification; EFS. status: event‐free survival status; OS. status: overall survival status; and OS. time: overall survival time. (B) Chord diagram showing KEGG pathway enrichment analysis based on turquoise module genes. (C) GO pathway enrichment analysis based on turquoise module genes. (D) Co‐expression network diagram of candidate genes in the turquoise module. Hexagon represents NB driver genes from the literature, circle represents machine learning‐derived prognostic biomarkers, and triangle represents genes shared by both. (E) Correlation between the number of connections of regulatory genes identified through GeneWalk analysis and the fraction of associated GO terms. (F) Venn diagram showing overlap between regulatory genes identified using GeneWalk and hub genes of the turquoise module identified by WGCNA. GO, gene ontology; KEGG, Kyoto Encyclopedia of Gene Genomes; NB, nueroblastoma; WGCNA, Weighted gene co‐expression network analysis.

To further refine prognosis‐related hub genes, co‐expression network analysis was performed using the WGCNA on the integrated set of selected prognostic biomarkers and NB driver genes. The WGCNA dendrogram identified five co‐expression gene modules (Supporting Information S1: Figure S1C), with the turquoise module demonstrating a significant correlation with overall survival time grouping (cor = −0.44, *p* value = 2e‐57, and Figure 2A). Pathway enrichment analysis was conducted on the turquoise module to identify pathways most significantly associated with survival. KEGG analysis revealed enrichment in pathways involved in cancer development, including the p53 signaling pathway, PD‐L1 expression and PD‐1 checkpoint pathway, and PI3K‐Akt signaling pathway (Figure 2B). GO analysis indicated significant enrichment in biological processes and molecular functions such as positive regulation of the cell cycle, spindle organization, and ubiquitin‐protein ligase binding (Figure 2C).

A co‐expression network of module genes most significantly associated with survival time was constructed using WGCNA. After filtering the top 30% of edges, the final network consisted of 163 nodes and 1860 edges (Supporting Information S1: Figure S1D). Hub genes were identified using the minimum spanning tree approach, as applied in previous study, and visualized using Cytoscape (Figure 2D) [20]. These hub genes, including *ALK*, *BRCA1*, *BRIP1*, and *CDC25 A*, emerged as both significant drivers in NB and critical network components. Further analysis of the turquoise module using the GeneWalk identified key regulatory and multifunctional genes, which demonstrated significant centrality within the network (Figure 2E, Supporting Information S1: Figure S1E). Notably, hub genes (such as *TP53*, *EGFR*, and *CDKN2A*) exhibited strong interconnectivity in the network, which suggests that they may play pivotal roles in regulating cancer progression and prognosis [40, 42].

Eleven prognostic related genes were identified via overlapping between the WGCNA‐derived hub genes and the GeneWalk analysis, including *AURKA*, *BLM*, *BRCA1*, *BRCA2*, *CCNA2*, *CHEK1*, *E2F1*, *MAD2L1*, *PLK1*, *RAD51*, and *RFC3* (Figure 2F). These genes demonstrated potential prognostic relevance and were identified as critical biomarkers. Notably, *BRCA1, BRCA2*, and *AURKA* played significant regulatory roles within the gene expression network and are strongly implicated in cancer onset and progression [43]. Notably, *RFC3* emerged as a novel prognostic biomarker for NB, which plays a crucial component of DNA replication factors [44]. Highly expressed *RFC3* has been reported to associate with a worse prognosis in head and neck squamous cell carcinoma and lung adenocarcinoma [45, 46]. However, its specific role in NB is remains to be elucidated.

In conclusion, the integrative analysis identified hub genes associated with NB prognosis and provided a potential biomarker for further functional studies.

### 3.3 Prognostic Significance of Hub Genes in NB

**FIGURE 3:**
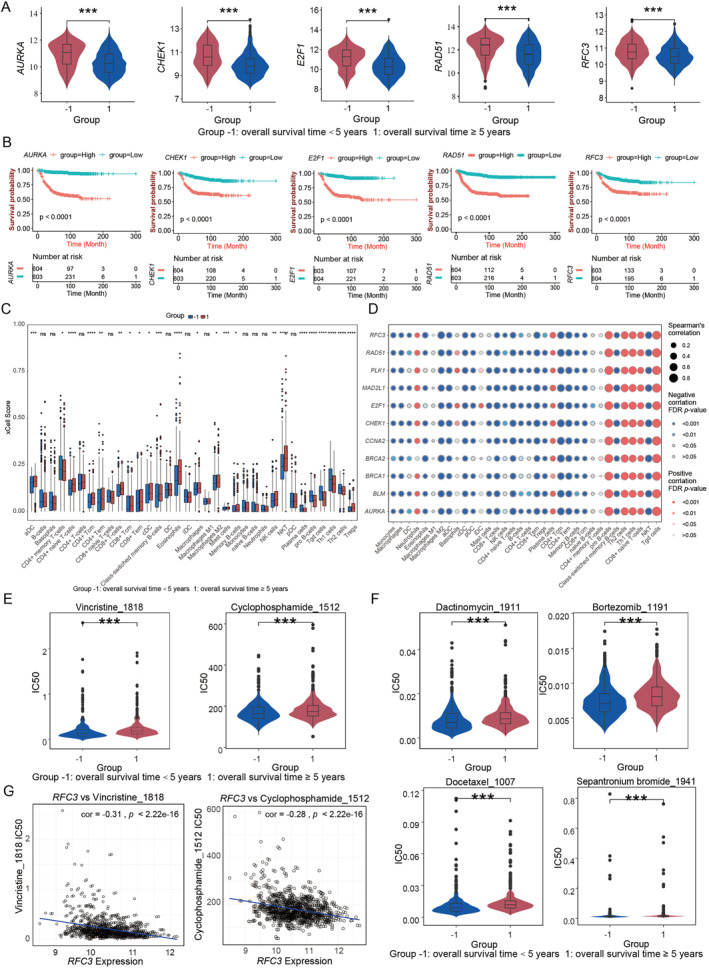
Downstream analysis of prognostic markers. (A) Violin plot showing expression differences in prognostic biomarkers between good and poor prognosis groups. Red: bad prognosis and blue: good prognosis. (B) The Kaplan–Meier curve showing survival probability differences between high‐ and low‐expression groups for prognostic markers. (C) Boxplot showing relative abundance distribution of immune cell types obtained from xCell analysis between good and poor prognosis groups. ****: *p* < 0.0001, ***: *p* < 0.001, **: *p* < 0.01, *: *p* < 0.05, and “ns” for no statistically significant difference. (D) Spearman correlation analysis between prognostic biomarkers and immune cell types. (E) Violin plot showing distribution of IC50 values for vincristine and cyclophosphamide between good and poor prognosis groups. (F) Violin plot showing distribution of IC50 values for dactinomycin, bortezomib, docetaxel, and sepantronium bromide between good and poor prognosis groups. (G) Correlation between *RFC3* gene expression and IC50 values of vincristine and cyclophosphamide. IC50: Half‐maximal inhibitory concentration.

To evaluate the prognostic potential of the 11 key genes identified, differential expression tests and survival analyses were conducted between patient groups with distinct overall survival outcomes. These survival results demonstrated that these prognostic markers were significantly upregulated in the poor prognosis group but exhibited lower expression levels in the good prognosis group (Figure 3A and Supporting Information S1: Figure S2A). Kaplan–Meier survival analysis further revealed that patients with high expression experienced significantly reduced survival time, whereas those with low expression showed prolonged survival time (Figure 3B and Supporting Information S1: Figure S2B). These findings suggest that the elevated expression of the identified prognostic biomarkers correlates with poorer outcomes, highlighting their potential roles in cancer progression. This pattern was consistent across several key genes, with previous studies supporting a significant association between high expression of *AURKA*, *CHEK1*, *E2F1*, and *RAD51* and adverse prognoses [47, 48, 49, 50] (Supporting Information S1: Table S2). Notably, high expressed *RFC3* was also linked to bad survival result, which suggest that *RFC3* might serve as a novel prognostic biomarker.

To evaluate differences in immune cell infiltration within the tumor microenvironment and their association with overall survival, immune‐related analyses were conducted. The xCell and MCP counter results indicated that natural killer (NK) cell scores were significantly higher in the favorable prognosis group (Figure 3C and Supporting Information S1: Figure S2D). This finding aligns with the former study of NK cells in directly eliminating tumor cells and regulating immune responses through the secretion of cytokines and chemokines [51]. Conversely, the xCell and the ESTIMATE results demonstrated that patients in the poor prognosis group had lower immune scores and higher tumor purity (Supporting Information S1: Figure S2C,E). Correlation analysis between immune cell infiltration and the prognostic markers revealed a significant positive association with neutrophils, plasma cells, and thymine‐DNA glycosylase cell (Tdg cell). In contrast, NK cells and CD8^+^ T cells exhibited notable negative correlations with these partial biomarkers, but dendritic cells (DC) is only negatively correlated with part of those biomarkers (Figure 3D). High NK cell infiltration is associated with favorable prognosis [51], which also identified our results. NK cells are negatively correlated with prognostic biomarkers, whereas elevated expression of these biomarkers is linked to poorer outcomes.

### 3.4 Therapeutic Target Potential of Hub Genes

In order to evaluate how these identified hub genes correlated to drug sensitivity in the patients with NB in both the favorable and unfavorable prognosis groups, a drug sensitivity analysis was performed. Drug sensitivity analysis of these hub genes revealed that the half maximal inhibitory concentration (IC50) of vincristine was lower than that of cyclophosphamide, which suggests that patients with NB could be sensitivity to vincristine treatment (Figure 3E). Furthermore, dactinomycin, bortezomib, docetaxel, and sepantronium bromide also exhibited low IC50 values, which suggests that they could be considered as potential therapeutic drugs for NB treatment (Figure 3F).

Correlational analysis between the expression levels of prognostic biomarkers and commonly used chemotherapeutic agents in the NB revealed hub genes associated to vincristine and cyclophosphamide (Figure 3G and Supporting Information S1: Figure S3A,B). Notably, the *RFC3* expression was negatively correlated with the IC50 values of both vincristine and cyclophosphamide. Higher *RFC3* expression was associated with lower IC50 values for these drugs, suggesting enhanced sensitivity in patients with the elevated *RFC3* expression. Given that vincristine and cyclophosphamide are key components of standard NB chemotherapy regimens [52], this relationship points to a potential molecular mechanism by which *RFC3* influences drug sensitivity. These findings suggest that the *RFC3* expression may serve as a valuable biomarker for predicting patient sensitivity to vincristine and cyclophosphamide, offering a basis for further optimizing personalized treatment strategies.

### 3.5 ScRNA‐Seq Analysis Suggests More T Cells Lead to Better Prognosis in the NB

**FIGURE 4:**
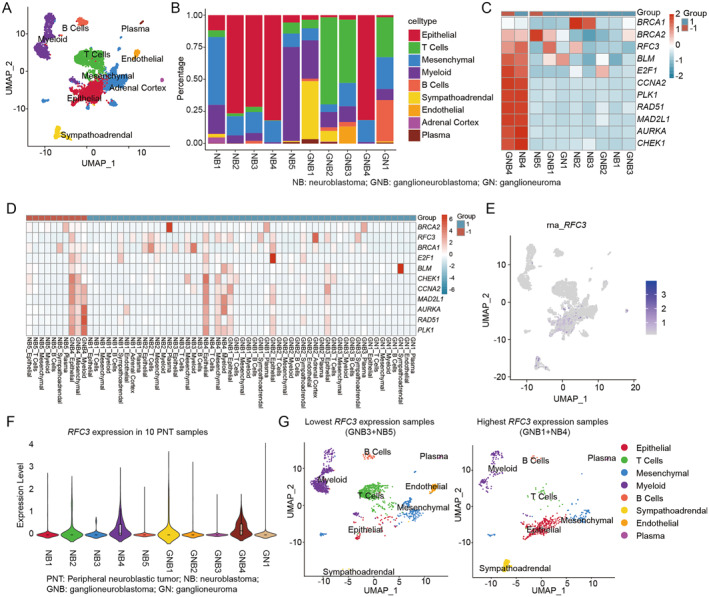
Single‐cell correlation analysis of prognostic biomarkers. (A) UMAP plot depicting 5358 total cells from 10 PNT samples, color‐coded by cell type. (B) Stacked bar chart showing cell‐type composition per sample, with cell counts normalized to 100%. Sample types are annotated at the top and patients are labeled along the *x* axis. (C) Heatmap showing average expression levels of prognostic biomarkers across PNT samples. (D) Heatmap showing average expression levels of prognostic biomarkers in different sample subgroups. (E) UMAP plot showing expression pattern of *RFC3* gene. (F) Violin plot showing *RFC3* expression levels across PNT samples. (G) UMAP plot showing distribution of two high‐expression samples and two low‐expression samples for the *RFC3* gene. GN, ganglioneuroma; GNB, ganglioneuroblastoma; NB, neuroblastoma; PNT, peripheral neuroblastic tumor; UMAP, Uniform Manifold Approximation and Projection.

To further explore the association between prognostic biomarkers at different cell subpopulations and related tumor microenvironment, we performed scRNA‐seq analysis of 10 patients with PNT, which include 5358 cells [13]. Based on the scRNA‐seq data, we identified nine cell types, which included epithelial, mesenchymal, myeloid, sympathoadrenal, endothelial, adrenal cortex, plasma, B cells, and T cells (Figure 4A). To assess correlation with clinical prognosis, patients of the scRNA‐seq were stratified into two groups based on overall survival: ≤ 3 years (marked as −1) and > 3 years (marked as 1). Analysis of cellular composition revealed significant heterogeneity in the PNT (Figure 4B). Patients with longer survival time exhibited a higher proportion of T cells, whereas those with shorter survival time showed a higher proportion of epithelial and myeloid cells (Supporting Information S1: Figure S4B,C).

Expression of prognostic biomarkers differed significantly between the two groups, with higher levels observed in ganglioneuroblastoma 4 (GNB4) samples from the shorter survival group, a trend consistently seen across key markers (Figure 4C). In the epithelial cell cluster, prognostic biomarkers exhibited elevated expression, with certain genes, such as *RFC3* and *CHEK1*, showing significantly higher levels in the shorter survival group compared to the longer survival group (Figure 4D,E, and Supporting Information S1: Figure S4D). Patients with low *RFC3* expression showed increased T cells and endothelial cells and decreased epithelial and sympathoadrenal cells (Figure 4F,G). *RFC3* expression was negatively correlated with T cell infiltration, consistent with the RNA‐seq findings. These results suggest that *RFC3* may influence cell subtype proportions within the tumor microenvironment and impact NB prognosis, highlighting its potential as a therapeutic target for future research.

## 4 Discussion

As the most prevalent solid tumor in infants, the NB exhibits remarkably diverse outcomes, ranging from spontaneous regression to aggressive and fatal disease [36]. Mosaicism is less common for single nucleotide variants (SNVs) than subchromosomal aberrations [37]. This heterogeneity contributes to inconsistent treatment responses, underscoring the necessity for in‐depth investigations into prognostic biomarkers that can inform diagnostic and therapeutic strategies [53]. Direct use of single cell data for biomarker prediction offers several advantages, including the ability to reveal tumor heterogeneity, capture rare cell populations, and enable personalized medicine [54]. However, identifying biomarkers in high‐dimensional, sparse, and noisy single‐cell datasets remains a significant statistical challenge [55]. In practice, combining bulk RNA data with single cell data may provide a more practical approach to biomarker discovery, leveraging the strengths of both methods while addressing their respective limitations. In this study, we proposed a comprehensive data‐driven approach to integrate bulk RNA‐seq and scRNA‐seq data for the identification of the NB prognostic biomarkers and their associations with the tumor microenvironment. By enhancing previous methodologies that incorporate prior network to biomarker discovery, we improved the framework for identifying hub genes, effectively isolating biomarkers with significant prognostic identification.

Given the substantial heterogeneity of NB, many studies have sought to identify clinically relevant biomarkers. For instance, Mossé et al. utilized a genetic homogeneity model to establish heritable *ALK* mutations as a primary cause of familial NB, highlighting *ALK* as a viable therapeutic target for this lethal pediatric malignancy [56]. Similarly, based on genomics and RNA‐seq analyses, former genomic researches identified *TERT* rearrangements as a novel therapeutic avenue for high‐risk NB and improved stratification [10, 57]. However, despite these advances, the prognostic value of single‐gene signatures in NB remains unstable and unreliable across diverse clinical scenarios. Here, we utilized a machine learning model and identified 11 key prognostic biomarkers for NB, including *AURKA*, *BLM*, *BRCA1*, *BRCA2*, *CCNA2*, *CHEK1*, *E2F1*, *MAD2L1*, *PLK1*, *RAD51*, and *RFC3*. Several of these prognostic biomarkers, including *AURKA*, *RAD51*, *CHEK1*, and *E2F1*, are consistent with previous reports, supporting their relevance in NB prognosis and progression [47, 48, 49, 50].

Notably, the *RFC3* was identified as a novel prognostic biomarker for NB in this study, which revealed significantly higher *RFC3* expression in the poor prognosis group, with elevated levels associated with markedly poorer overall survival. The *RFC3,* as a crucial component of the DNA replication machinery, has been implicated in various cancers [44], such as head and neck squamous cell carcinoma and lung adenocarcinoma [45, 46]. Our findings also demonstrated that patients in the favorable prognosis group exhibited higher NK cell scores in the bulk RNA profiles. As cytotoxic lymphocytes of the innate immune system, NK cells are capable of eliminating virus‐infected and cancerous cells [58]. This is consistent to the previous study of Melaiu et al., which shows the NK cells favorable to cancer prognosis [51]. Similarly, our previous study indicated that the neurogenic subtype, characterized by increased NK cell infiltration, correlated with the most favorable prognosis [59]. These observations suggest that the *RFC3* expression and the NK cell activity may serve as valuable targets for developing therapeutic strategies to enhance patient management and survival outcomes.

This study has several limitations. Without biological validation in our current research, our findings need further in‐depth study. Meanwhile, as gene expression may be influenced by mutation, miRNA regulation, and protein‐level translation, which incorporated multiomics data for identifying valuable biomarkers [36], more efficient machine learning models for integrating multiple omics data of cancer could help to discovery more valuable biomarkers.

## 5 Conclusion

In conclusion, this study identified several prognostic biomarkers for the NB and found the *RFC3* emerging as a novel key prognostic biomarker. These results provide a potential biomarker and data integration pipeline for further functional validation and biomarker discovery, which could be useful in personalized treatment strategies in cancer biomarker discovery.

## Author Contributions

**Shuxin Tang:** methodology, bioinformatics and data analysis, writing – original draft preparation, writing – review and editing. **Jinhua Fan:** conceptualization, methodology, writing – original draft preparation, writing – review and editing. **Yupeng Cun:** conceptualization, methodology, bioinformatics and data analysis, investigation, writing – original draft preparation, writing – review and editing.

## Ethics Statement

As all data analyzed in this study were obtained from publicly available datasets, no ethical approval was required for this study.

## Consent

All the authors have read the manuscript and agreed to its publication.

## Conflicts of Interest

The authors declare no conflicts of interest.

## Supporting information

Supporting Information S1

## Data Availability

The code generated during this study will be available upon publication of the article. The gene expression data of patients with NB were obtained from the GEO database (GSE49710) and EMBL‐EBI database (E‐MTAB‐1781). Single‐cell RNA‐seq data of patients with NB were obtained from the GEO database (GSE192906).
