# Supplementary material for: Identification of Prognostic Biomarkers in Gene Expression Profile of Neuroblastoma Via Machine Learning
Source: Pediatr Discov. 2025 May 27;3(2):e70009. doi: 10.1002/pdi3.70009 (PMC12258108; doi:10.1002/pdi3.70009)
Supplement: Supplementary file 1 — Supporting Information S1 [file PDI3-3-e70009-s001.docx]

**Supporting Tables and Figures**

**Table S1. 46 driver genes of NB from literature.**

| *ALK* | *BRCA1* | *CREBBP* | *KNTC1* | *ODC1* | *SFN* |
| --- | --- | --- | --- | --- | --- |
| *APC* | *BRIP1* | *DDX1* | *KRAS* | *OS9* | *SHANK2* |
| *ARID1A* | *CCND1* | *EBF1* | *MDM2* | *PHOX2B* | *SLX4* |
| *ARID1B* | *CDC25A* | *FGFR1* | *MYCN* | *PTPN11* | *SMARCE1* |
| *ATM* | *CDK12* | *FGFR4* | *NBAS* | *PTPRD* | *TERT* |
| *ATRX* | *CDK4* | *HMGA2* | *NF1* | *RAD51C* | *TP53* |
| *AXIN2* | *CDKN2A* | *HRAS* | *NME1* | *RBMS3* |  |
| *BRAF* | *CDKN2B* | *KIT* | *NRAS* | *SDHA* |  |

**Table S2. Reported research records of 11 key prognostic biomarkers for the 11 key prognostic biomarkers**

| Gene symbol | Tissue specificity | Documentation of prognostic biomarker for other cancer | Documentation of prognostic biomarker for NB |
| --- | --- | --- | --- |
| *AURKA* | Lymphoid tissue,  Testis | Bladder urothelial carcinoma,  Kidney chromophobe,  Kidney renal clear cell carcinoma,  Kidney renal papillary cell carcinoma,  Liver hepatocellular carcinoma,  Lung adenocarcinoma,  Pancreatic adenocarcinoma | A prognostic marker |
| *BLM* | Bone marrow,  Lymphoid tissue,  Salivary gland | Hepatocellular carcinoma,  Lung adenocarcinoma,  Rectum adenocarcinoma | A prognostic marker |
| *BRCA1* | Low tissue specificity | Hepatocellular carcinoma,  Lung adenocarcinoma,  Rectum adenocarcinoma | Not prognostic  (A therapeutic target) |
| *BRCA2* | Bone marrow,  Lymphoid tissue,  Testis | Not prognostic | A prognostic marker |
| *CCNA2* | Bone marrow,  Lymphoid tissue,  Retina | Kidney chromophobe,  Renal clear cell carcinoma,  Renal papillary cell carcinoma,  Hepatocellular carcinoma,  Lung adenocarcinoma,  Pancreatic adenocarcinoma | Not prognostic  (A potential target) |
| *CHEK1* | Bone marrow,  Lymphoid tissue,  Seminal vesicle | Renal clear cell carcinoma,  Hepatocellular carcinoma,  Lung adenocarcinoma,  Pancreatic adenocarcinoma | A prognostic marker |
| *E2F1* | Bone marrow | Kidney chromophobe,  Renal clear cell carcinoma,  Renal papillary cell carcinoma,  Hepatocellular carcinoma,  Pancreatic adenocarcinoma | A prognostic marker |
| *MAD2L1* | Bone marrow,  Lymphoid tissue | Renal clear cell carcinoma,  Hepatocellular carcinoma,  Lung adenocarcinoma | Not prognostic  (A progression-related essential gene) |
| *PLK1* | Bone marrow,  Lymphoid tissue,  Testis | Kidney chromophobe,  Renal clear cell carcinoma,  Renal papillary cell carcinoma,  Hepatocellular carcinoma,  Lung adenocarcinoma,  Pancreatic adenocarcinoma | A prognostic marker |
| *RAD51* | Lymphoid tissue,  Testis | Renal clear cell carcinoma,  Renal papillary cell carcinoma,  Hepatocellular carcinoma,  Lung adenocarcinoma,  Pancreatic adenocarcinoma | A prognostic marker |
| *RFC3* | Low tissue specificity | Renal clear cell carcinoma,  Hepatocellular carcinoma,  Lung adenocarcinoma,  Rectum adenocarcinoma | No reports related to NB |


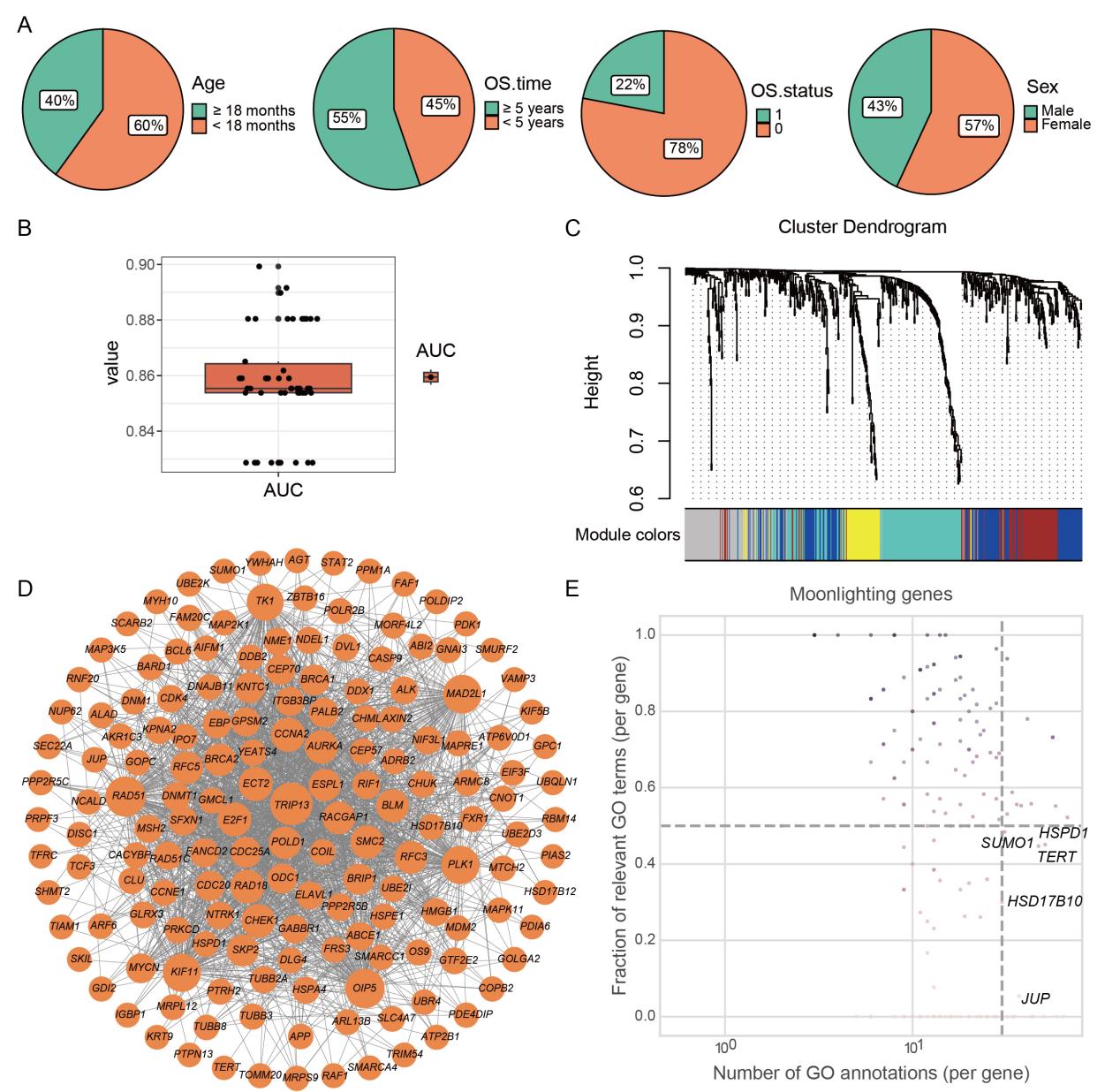


**Supplementary Figure S1.** (A) Distribution of key clinical characteristics in the study cohort, including: (1) age, (2) overall survival (OS) time, (3) OS status, where OS status = 1 indicates patient death and OS status = 0 indicates patient survival, and (4) sex. (B) Distribution of AUC values during machine learning model training. (C) Hierarchical cluster dendrogram of five gene co-expression modules. (D) Co-expression network diagram of candidate genes in turquoise module, with larger circles indicating genes with more connections. (E) Relationship between the number of GO annotations of moonlighting genes obtained through GeneWalk analysis and the fraction of relevant GO terms.


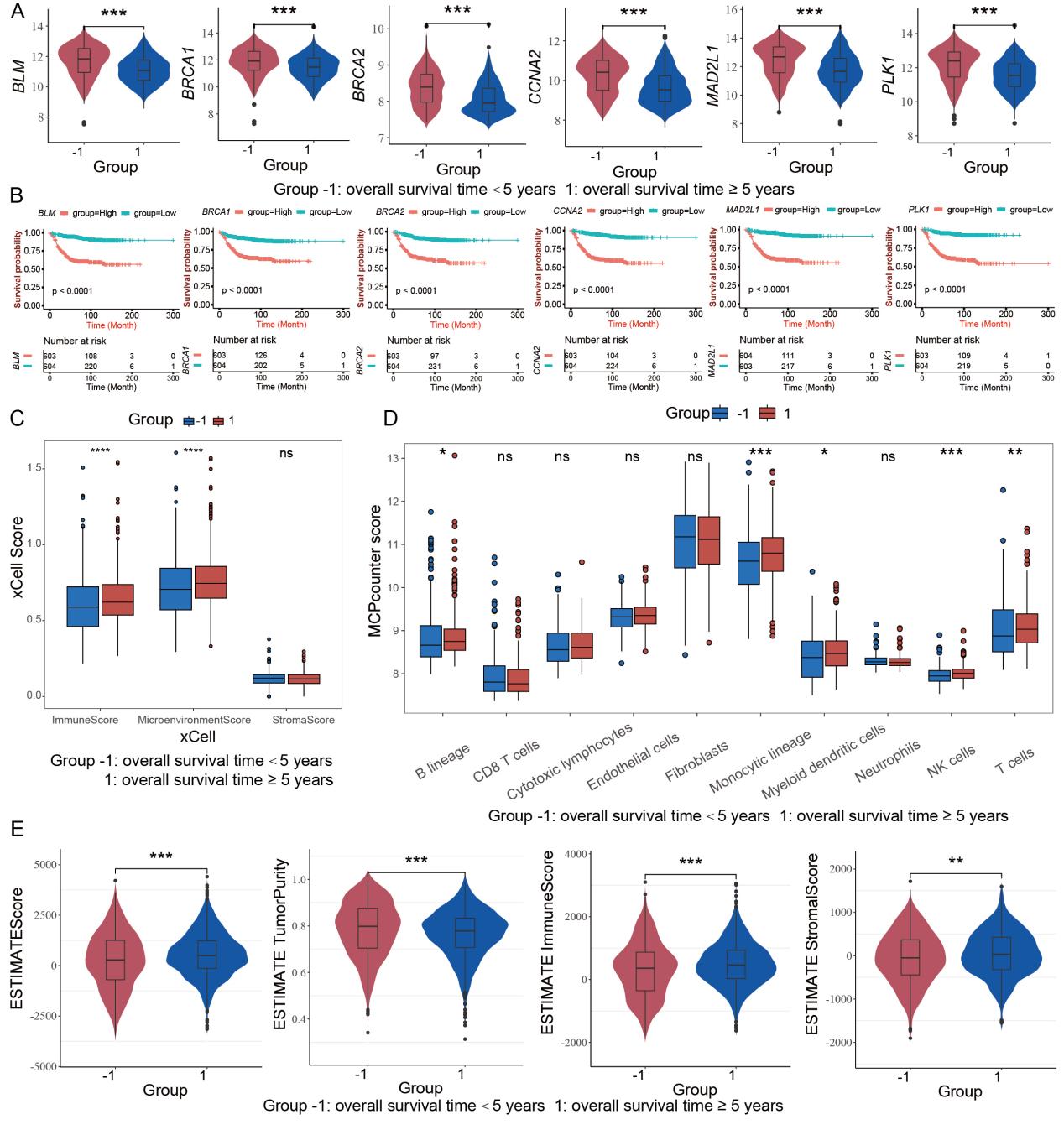


**Supplementary Figure S2.** (A) Violin plot showing expression differences in prognostic biomarkers between good and poor prognosis groups. (B) Kaplan-Meier curve showing survival probability differences between high- and low-expression groups of prognostic markers. (C) Differences in ImmuneScore, MicroenvironmentScore, and StromaScore between good and poor prognosis groups based on xCell analysis. ****: *p* < 0.0001, "ns": no statistically significant difference. (D) Differences in MCPcounter scores in different immune cell types between good and poor prognosis groups based on MCPcounter analysis. ***: *p* < 0.001, **: *p* < 0.01, *: *p* < 0.05, and "ns" for no statistically significant difference. (E) ESTIMATEScore, ESTIMATE TumorPurity, ESTIMATE ImmuneScore, and ESTIMATE StromalScores between good and poor prognosis groups based on ESTIMATE analysis. ESTIMATE: Estimation of Stromal and Immune Cells in Malignant Tumor Tissues Using Expression Data.


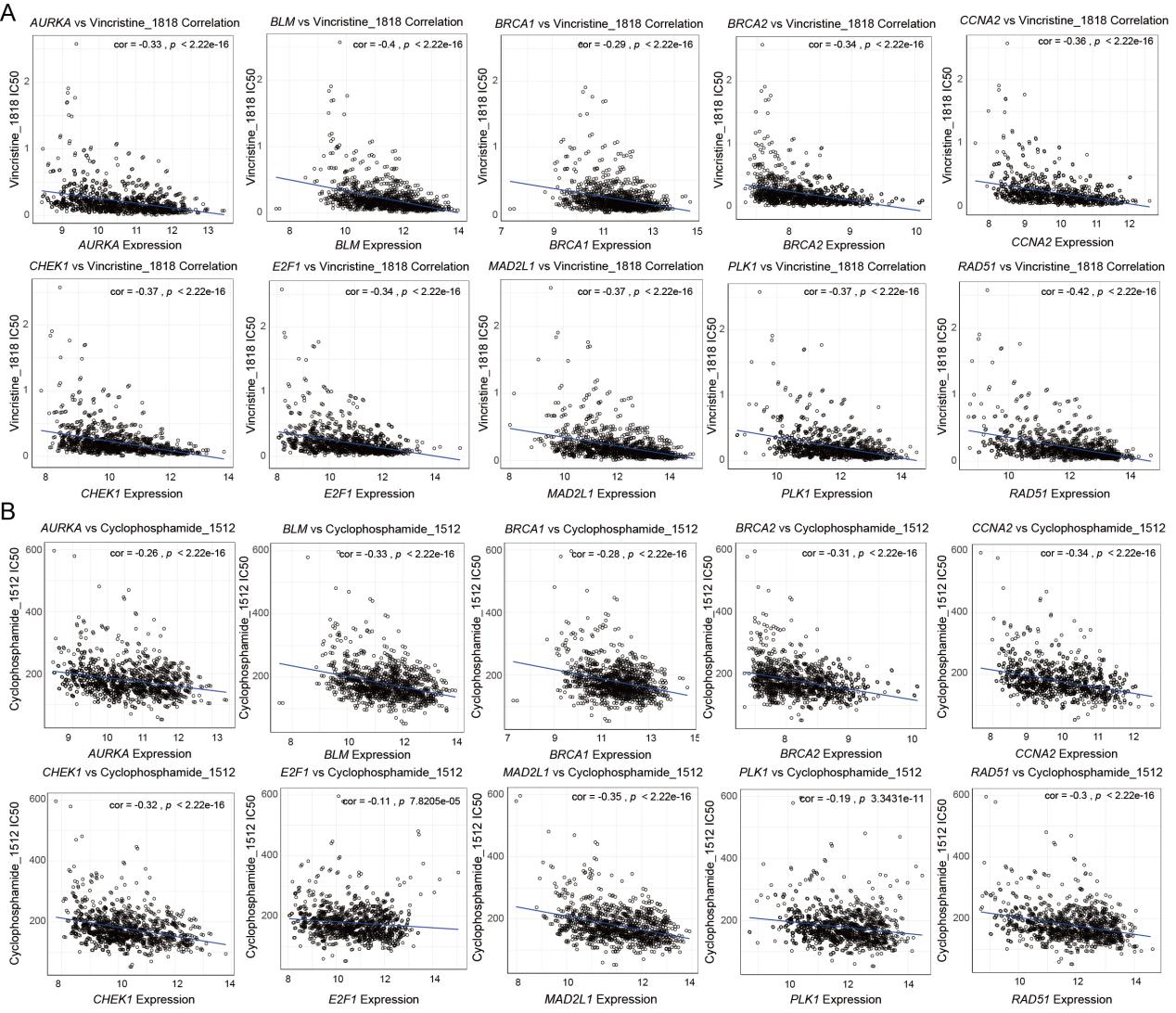


**Supplementary Figure S3.** (A) Correlation between expression of prognostic biomarkers and IC50 values of vincristine. (B) Correlation between expression of prognostic biomarkers and IC50 values of cyclophosphamide.


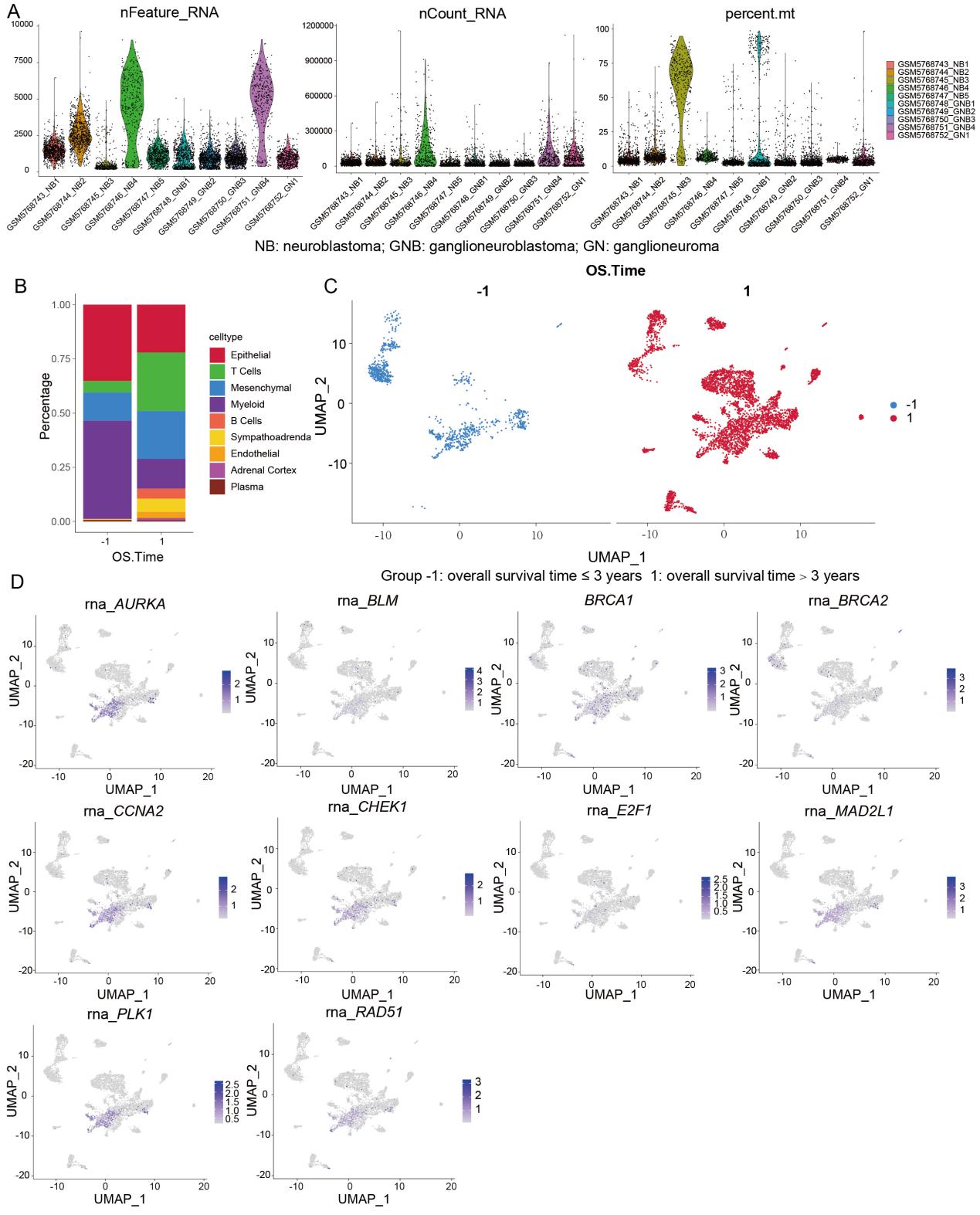


**Supplementary Figure S4.** (A) Violin plot showing distribution of RNA features, RNA counts, and mitochondrial percentage across different sample groups. (B) Proportional graph showing proportion of various cell types between good and poor prognosis groups (overall survival time ≤ 3 years, marked as -1; overall survival time ＞ 3 years, marked as 1). (C) UMAP plots showing distribution of good and poor prognosis groups. (D) UMAP plots showing expression of prognostic biomarkers. UMAP: Uniform Manifold Approximation and Projection.
